# Recognising and responding to clinical deterioration in acute hospitals. Recording patient-reported wellness after Martha's rule

**DOI:** 10.3389/frhs.2026.1854717

**Published:** 2026-08-26

**Authors:** Christian Peter Subbe, Lorelei Jones, Aimee Banfield, Sahla Siji, Lakshmipriya Munduchirayil Ajimon, Catherine Phythian, Adama Kaba

**Affiliations:** 1 Department of Medicine, Ysbtyty Gwynedd, Bangor & Advanced Internal Medicine, Woodlands Hospital, Singapore, Singapore; 2 North Wales Medical School, Bangor University, Bangor, United Kingdom; 3 Health Sciences, Bangor University, Bangor, United Kingdom

**Keywords:** digital health, martha's rule, patient safety, patient wellbeing, rapid response system (RRS), rapid response team, vital signs

## Abstract

**Background:**

Early recognition of clinical deterioration is central to patient safety, yet preventable harm still occurs. UK policy emphasizes listening to patients and systematically capturing patient-reported wellness.

**Aims:**

The aim of this study was to understand current practice for including patient and family voices in recognising and responding to deterioration, and to assess the feasibility of a structured approach to including patient-reported wellness as part of routine clinical monitoring.

**Methods:**

A mixed-methods feasibility study of a structured approach to capturing patient assessments of wellness (the Patient Wellness Scale) across four medical wards in an acute hospital in Wales.

**Findings:**

Healthcare staff routinely ask patients how they are feeling but documentation is inconsistent and fragmented. Testing the Patient Wellness Scale in a real-world clinical setting found it was not accessible to patients with speech and language difficulties, cognitive impairments, dementia and those who were critically unwell. In the context of staff shortages, temporary staffing and a task-based approach to care there is a risk that the Patient Wellness Scale could be implemented mechanistically.

**Conclusions:**

How patients are feeling is widely recognised as clinically meaningful but remains poorly formalised as a safety signal. A brief, structured Patient Wellness Scale embedded within routine observations could externalise tacit knowledge, support escalation across hierarchies, and align practice with national policy. However, there are potential unintended consequences. Our research identifies important considerations for policy makers and organisations implementing national policy requirements for patient-reported wellness.

## 1 Introduction

Recognising and responding to hospital patients who are deteriorating is a concern for healthcare systems around the world. In the UK, failure to adequately recognise and respond to clinical deterioration is a major cause of harm to patients in hospital (1, 2). One study found that up to 26% of preventable deaths in hospital are due to failures in monitoring patients (3).

Recent initiatives in NHS England and Wales are aimed at improving the way patients and their families are involved in patient safety, clinical monitoring and escalation (4, 5). These initiatives have been introduced in response to high profile cases where patients have died following delays in escalation of care, despite the patient or the family expressing concerns to healthcare staff. This paper reports from a feasibility study of a standardised approach to including patient-reported wellness in clinical monitoring in an acute hospital in Wales. The aim of the study was to understand how healthcare staff currently include patient voices in clinical monitoring and to assess the feasibility of a structured approach in clinical settings.

Failings in care related to recognising and responding to clinical deterioration have a significant impact on patients, families, healthcare staff, and organisations. In addition to avoidable suffering, disability and deaths, the burden for patients, families, and society extends to care needs beyond hospital and loss of income of patients and their carers (6). Adverse events also have an emotional impact on staff (7). Financial costs to the organisation also accrue from increased workload, litigation and length of stay (6, 8).

The case of Martha Mills, a teenager who died at Kings College Hospital in London, following an injury to her pancreas, underlined the importance of listening to the concerns of patients and their families who often know when care needs to be escalated. In Martha's case, there were delays in escalation of care, despite her parents voicing concerns that she was deteriorating. The case led to increased policy focus on listening to patients, families and carers in England and Wales (4, 5). Part of the policy is a requirement for NHS organisations to ask patients, at least daily, how they are feeling, and if they are getting better or worse, and to act on this information in a structured way. It is not known how patient-reported wellness is currently captured in hospitals in Wales and how to best implement the recommendation or how to optimise impact.

Patient harm can be reduced by timely treatment (9). However, recognising and responding to clinical deterioration involves a complex sequence of activities, and many different actors and technologies (10, 11). The process is made up of multiple components, such as monitoring vital signs, recording information, making calculations, interpreting information, communicating to other members of the team, requesting and waiting for specialist expertise, deciding an effective management plan and implementing the plan. Problems can occur at any “link” in the preventive “chain” (12).

Patients can participate in escalation of care (13–15) but the context in which this is most effective is not known. Previous research has highlighted challenges to patients' raising concerns (16), suggesting that normalisation of the process of reporting deterioration requires further attention. Few measures to capture patient wellness have been tested in hospital settings. The aim of this study was to understand current practice and to test an approach to including patient-reported wellness as part of routine clinical monitoring.

### 1.1 The patient wellness scale

Martha's rule introduced a requirement for hospitals to introduce a 'structured approach to obtain information relating to a patient's condition directly from patients and their families at least on a daily basis' (4). One way to do this is to use the Patient Wellness Scale.

The Patient Wellness Scale is taken from the Patient Wellness Questionnaire (17). It asks “How are you feeling compared to the last time we asked you?” with the following response categories: “Much worse, worse, the same, better, much better.” The Patient Wellness Questionnaire was evaluated in a sample of 103 patients (mean age 66, age range 39–90, 62% male) (18). When patients reported feeling “much worse” or “worse”, their own assessment of their wellness (on a scale of 1 to 10) was associated with physiological markers of deterioration as measured by the National Early Warning Score (NEWS). This association was significant at both the subsequent observations (2–6 h afterwards) and 24 h afterwards. While healthcare staff might often ask a patient “how are you feeling?” the Patient Wellness Scale ascribes a numerical value and enables analysis of trends. In this way it potentially offers a systematic and standardised approach to incorporating patient-reported wellness into clinical monitoring and has been recommended as the preferred tool for the implementation of Martha's rule (19)

This paper explores the feasibility of implementing the Patient Wellness Scale as part of routine vital sign monitoring in a single acute care hospital in Wales. We aimed to answer the following questions:

1. How do staff currently view, capture and act on patient-reported wellness?
2. How do staff interact with the Patient Wellness Scale when added to digital vital sign recording in the context of everyday work?
3. What are the implications for the potential for the patient Wellness Scale to improve patient care in the described setting and what might be the unintended consequences?

## 2 Methods

The paper reports from the first part of an evaluation of the Patient Wellness Scale in an acute hospital in Wales. The aim was to (1) understand current context and practice and (2) assess the feasibility of the Patient Wellness Scale in a real-world clinical setting. The study design followed established guidance for evaluating healthcare improvement initiatives (20). In the early phases of innovation development, the guidance recommends testing in a small number of clinical settings using qualitative methods, such as interviewing and observing clinical teams. These methods were used to understand how the Patient Wellness Scale is used in practice, suggest updates to the underlying theory of how the Patient Wellness Scale will bring about improvements in care and outcomes, and identify unintended consequences.

### 2.1 Conceptual background

The design of the study draws on sociotechnical perspectives that view patient safety as an emergent property of the interaction of health information technology with people, organisations and the broader social, cultural and political environment (21). This perspective recognises that technologies often fail, not as a result of the technology itself, but as a result of the complexities of using them in real-world contexts. It is therefore important to understand how the social and organisational context shapes the use and effects of technology. In this study we are interested in a what happens when a new question (the Patient Wellness Scale) is added to the electronic device for recording patient vital signs. Our theoretical assumptions are drawn from an evidence-based framework developed by Cressell et al. (21) for evaluating health information technology implementations: The Technology, People, Organization and Macroenvironmental factors (TPOM) framework integrates exiting evidence-based evaluation frameworks for Health Information Technology and is based on findings from formative evaluations of the implementation of three Health Information Technology projects in the UK NHS (electronic patient record, electronic prescribing, and clinical decision support systems). We therefore designed the evaluation to enable our analysis to account for the influence of the social, organisational and broader political context, and the way staff used the Patient Wellness Scale as part of every-day work. We used a combination of methods as follows:

To understand current context and practice:

1. A review of medical notes to understand how information about patient's assessment of their wellness was currently recorded
2. Focus groups with healthcare staff to understand staff views and experiences of asking patients how they were feeling, and more generally, of clinical monitoring in the context of their everyday work
3. Ward observation of everyday clinical care including clinical monitoring

To assess the feasibility of the Patient Wellness Scale in everyday clinical practice:

1. A test of the verbal version of the Patient Wellness Scale in patients across four medical wards in an acute hospital
2. Ward observation as staff interact with the electronic version of the Patient Wellness Scale in everyday clinical settings

### 2.2 Setting

The setting for the study were four wards in an acute hospital in Wales, including the Acute Medical (Admissions) Unit, an acute respiratory ward with a respiratory high care area, a general medical ward with a focus on gastroenterological conditions, and a ward with a focus on re-enablement. Patients had a broad range of acute and chronic medical conditions including pneumonia, skin infections, myocardial infarction, heart failure, diabetes, epilepsy, Parkinsons disease, asthma, Chronic Obstructive Pulmonary Disease, falls, frailty syndromes, and complications of cancer.

### 2.3 Data collection

#### 2.3.1 Understanding current context and practice

(i) Review of medical notes

On the study wards all patient files were reviewed on four consecutive Fridays in November and December 2024 (all documentation for 448 patients). Nursing and medical records were reviewed for documentation of wellness and barriers to communication. Vital sign charts were screened for evidence of abnormal vital signs. Data was collected by medical students (AB, SS, LMA, CP, AK), who were undertaking a healthcare improvement project as part of the student selected component of the medical school curriculum. Data was collected into an excel spreadsheet including data on the wellness scale, quotes from patients made during the collection of responses to the wellness scale and values of the National Early Warning Score (NEWS) at time of the data collection and from the previous day.

(1) Ward observation

Observation (LJ) was used to understand pre-implementation clinical practice and to capture clinical monitoring in the context of every-day working life. The process followed the procedure used in previous studies (22, 23). Observation involved a combination of shadowing healthcare staff (including a senior doctor, a nurse and a healthcare assistant) to gain insight into their working day and observing ward routines from a chair positioned in one of the bays, either at the far end, below and slightly to one side of a TV, or just inside the door that led to the bay from the corridor. The aim was to have a view of the bay but without infringing on patients' dignity or personal space or interfering with the work of staff. Observation (90 h) was undertaken in either 3- or 6 h blocks and included a mix of day, night, and weekend shifts. LJ made contemporaneous notes in a notebook during ward observations.

(1) Staff focus groups

Five focus groups were held in January 2025. Four groups were held with a total of 26 nursing staff (25 were women). Groups were made up of a mix of grades and roles (11 nurses, 9 health care assistant, 2 clinical support workers, 2 house keepers, 1 ward manager, and 1 student nurse). One group was held with 9 resident doctors (7 were women). Staff were asked about their views and experience of monitoring patients for signs of deterioration, asking patients how they were feeling, and suggestions for areas of improvement. The focus groups were facilitated by AB, SS, LMA, CP, AK, and LJ. None of the interviewers were previously known to the participants of the focus groups and were not employed by the hospital. Recordings were made on a dedicated Dictaphone and transcribed.

Data from focus groups were analysed using thematic content analysis (24). This approach analyses the content of the data to categorise recurrent “themes”. The aim was to report the key elements in participants’ accounts and relate these to our research questions to identify, understand and explain salient issues. Our process was interpretive, comparative and iterative, using a combination of inductive and deductive forms of inference based on a close reading of transcripts alongside fieldnotes from observation, recent academic literature, and national reports on clinical deterioration. The procedure was as follows: Each author independently read and coded each transcript. Here “coding” involved marking up the content of what is being said, adding labels to sections of text, making short notes in the margins, and writing longer analytical memos in a notebook or a separate document. Marking up and labelling text was based on instances of repetition, relevance to the research question, and significance to broader literature and policy debates. We then met in person as a team to discuss and synthesise our impressions, reflections and ideas, and distil and expand our interpretive categories using a flip chart. We refined and developed our findings through further discussion, and through writing, sharing and rewriting drafts of the manuscript.

#### 2.3.2 Assessing the feasibility of the patient wellness scale in clinical settings

##### (i) Testing a verbal version of the Patient Wellness Scale

The sampling frame consisted of all patients on the four wards on four consecutive Friday afternoons in November and December 2025 (*N* = 448). Patients were interviewed where clinical teams agreed, patients were available and gave verbal consent to take part in the survey. Patients were asked whether they felt much better, a bit better, the same, a bit worse or much worse than the previous day. The value of the National Early Warning Score (NEWS) at the time of interview and from the previous 24 h were charted.

##### (1) Observing how clinical staff interact with the integration of the Patient Wellness Scale into existing digital monitoring

As part of service improvement, the hospital tested the addition of the Patient Wellness Scale to existing digital platforms. The Patient Wellness Scale was added to the existing spot-check monitors (MP5SC, Philips, Böblingen, Germany). Spot-check monitors are used to record blood pressure, temperature, oxygen saturation, respiratory rate, heart rate, and alertness of the patient. Individual scores are used to calculate NEWS. These readings are then transferred to a paper chart attached to a clipboard and hung on the side of the bed. Ward observations were undertaken across two wards on two consecutive Friday afternoons in March 2026 to capture how staff interacted with the Patient Wellness Scale (AB, SS, LMA, CP, AK).

### 2.4 Research team

It is important to be transparent about the make-up of the research team and to reflect on how this might influence the generation and interpretation of data. The first author is a consultant physician who led the quality improvement project. He has previous experience of implementing standardised digital approaches to improving patient care. There is therefore a potential for confirmation bias. As a senior member of the hospital hierarchy he might have also influenced the accounts and behaviour of staff due to social norms that shape dialogue, and concerns that staff may have about how revelations might impact on working life. The first author was therefore not involved in the qualitative data collection. The second author is a social scientist with an interest in how the use and effects of patient safety interventions are shaped by social processes and contexts. The remaining authors are medical students who were undertaking a healthcare improvement project as part of the student selected component of the medical school curriculum. There is therefore a mix of insider and outsider positioning, interests, and perspectives on digital interventions in healthcare.

### 2.5 Ethics

The study followed the NHS Health Research Authority (HRA) policy framework for health and social care research. It was assessed to be service improvement that did not require Research Ethics Committee Review because it was designed to observe the existing delivery of care and deliver improvements to the service through an intervention that is new in the context (see Supplementary Appendix S1). The study was therefore registered as a quality improvement project with the Health Board research and development office. A screening tool adapted from the Health Care Quality Improvement Partnership was used to review the study for ethical questions (25). We also used published guidance to design and conduct the evaluation (26). The process for obtaining appropriately informed voluntary consent from participants (staff and patients) followed UK Health Research Authority (HRA) guidance. A participant information sheet was provided to each potential participant based on HRA templates and consent was recorded verbally with the option to “opt out” (18), either by not contributing to the focus group (staff) or declining not to answer the PWS (patients). All data was anonymised and stored securely on the NHS network in line with the Data Protection Act 2018 and UK General Data Protection Regulations.

## 3 Findings

### 3.1 Understanding current context and practice

#### 3.1.1 Monitoring subjective wellness is routine but implicit

Across all staff groups, monitoring subjective wellness was described as routine and informal. Doctors, nurses and healthcare assistants all reported asking patients “how are you feeling?” but said that this was embedded in everyday interactions rather than undertaken as a discrete task. The following conversation is from a focus group:

Nurse 1: I do that every morning…
Nurse 2: Yeah, you automatically …
Healthcare Assistant 1: I go in and I say “good morning how are you feeling today?”
Nurse 2: Introduce yourself and say “how are you feeling?” (Nurse)
Nurse 3: You do ask them, yeah “how you feeling?, better now?” yeah
Healthcare Assistant 2: A lot of people will tell you, obviously dementia patients they can't, most people they'll tell you how they feel. And if they feel any worse I think they'll say

Doctors described documenting wellness to support clinical reasoning. For example, one resident doctor explained that “I record it because it is how I can show that what I have done has actually helped. It's a justification for why you have chosen the plan that you do.”

#### 3.1.2 Time pressure shapes interaction with patients

Severe time pressure dominated accounts of daily work, and this influenced how often staff asked patients how they were feeling and if and when this was recorded. For nurses time pressure resulted from staff shortages. The workforce model was for one nurse and one healthcare assistant per each six-bed bay. However, workforce shortages meant that staff were routinely responsible for two bays. In the following example a nurse is saying that while she does ask patients how they are feeling she may not get a chance to add this information to the nursing record until the end of the shift:

“I try to write it in my documentation, but I don't get a chance to sit down and write until 6 o'clock in the evening. I try to scribble it on some scrap paper and add it all in later but…yeah” (Nurse)

The priorities for nursing staff were the medication round and completing the electronic nursing record. On one ward the pressure to complete the medication round meant that nurses did not participate in the medical ward round. The medical ward round involved the consultant, resident doctors and medical students talking to each patient in turn. These conversations developed a shared situational awareness and understanding of the management plan. Crucially, the situational awareness incorporated the views of patients and, if present, family members, on how the patient was feeling, any problems, and their preferences and priorities. Nurses relied instead on transferring information from the medical notes into the nursing record. This process was often delayed or incomplete, due to difficulties understanding handwriting or medical notes missing from the storage trolley because they were being used by another member of staff.

Both nurses and healthcare assistants were employed on 12 h shifts. Nurses estimated that they spent about 80% of their time on documentation and this estimate was consistent with what was recorded during ward observations. Nurses spent very little time in proximity to the patients in the bay. Most time was spent completing the electronic nursing record, either seated at a desk in a central area, or in the corridor outside the bay where the medical notes were stored in a trolley. Here nurses positioned themselves to transfer information from the medical notes, which were stored in A4 binders, into the nursing record using a tablet (hand-held electronic device). In the periods of observation, documentation was never able to be completed during the shift, so nurses used their breaks, taking the tablet into the break room, and stayed back after a shift had ended, sitting at a desk computer in a central area, until the documentation was completed.

Documentation related to a long list of mandatory reporting requirements, including catheter bundles, PVC bundles, fluid balance, stool charts, pressure sore bundles, and falls risk. The mandatory reporting was audited weekly, and, as one nurse said in a focus group, “if they are not done you get in trouble”. Electronic fields had a time clock and performance, in terms of completing the documentation, was colour coded—red, amber or green. The information on performance was shared with a scrutiny committee.

The medication round and documentation comprised most of the nurses' work. As a result, clinical observations were delegated to healthcare assistants, to be completed alongside their other tasks, such as stripping and making beds and personal care (washing and helping patients use the toilet).

“So I've got 11 patients and it's just trying to do the Med rounds in the morning. It's just I don't get time to do the Obs as well.” (Nurse)

Some healthcare assistants were permanent members of the ward team, and many were very experienced. However, many were temporary staff (bank or agency staff) with unknown or variable skills and training in clinical observation. When temporary staff were not trained in observations this further increased the workload for the healthcare assistants who were trained. Other common problems raised in focus groups related to recording patient observations included missing equipment, incorrect use of equipment, charts where observations were missing or calculations were incorrect, and healthcare assistants who did not have the knowledge, skills or confidence to interpret the data and communicate concerns to nursing staff.

Under severe time pressure the priority, for both nurses and healthcare assistants, was efficient completion of tasks. Clinical monitoring was often completed in silence. It was only the pain scale that required the nurse to speak to the patient. If a patient could not be woken by gentle prompting, they were left sleeping. Three observations (blood pressure, temperature, oxygen saturation) were read off a screen which was positioned in such a way as to require the healthcare assistant to face away from the patient. The respiratory rate was recorded by watching the patient's chest. This was often done for 15 s then multiplied by 4 to get the reading for a minute. Only alertness scale required the healthcare assistant to look at the patient's face.

Time pressures also influenced the way doctors interacted with patients and asked them how they were feeling. Some doctors reported avoiding open-ended conversations in acute areas due to throughput pressures. In the following extract a doctor is suggesting that the practice of “corridor care”, that is locating patients in corridors when there is no more appropriate space available, is unlikely to be conducive to an honest account due to the lack of privacy:

“You can still ask them, but you know that you are not going to get the answer you would get if you were in a private room or with a curtain around them.” (Doctor)

#### 3.1.3 Knowing the patient matters

Recognition of deterioration was frequently described as experiential and relational. Healthcare Assistants and Clinical Support Workers, in particular, emphasised their ability to detect subtle changes through prolonged contact:

“I’ve been here 32 years so you tend to know. I could walk into that bay, and I would think “my patient's not looking very well. Let's do something about it.”” (Healthcare Assistant)

Doctors echoed this, highlighting the importance of continuity:

“Sometimes it is the only sign that they are not going the right way. Sometimes their bloods will look ok and the NEWS will be 0 but they look awful.” (Resident Doctor)

However, staff turnover, temporary staff, rotational training, and frequent patient moves undermined this safety mechanism:

“The […] ward, does get a lot of locum staff and things so there isn't much continuity there and you don't have that same person seeing them every day to compare.” (Resident Doctor)

#### 3.1.4 Documentation is fragmented and information about subjective wellness is hard to find

Information about how patients are feeling was hard to find in written documents. Of the 448 patients in our sample, we located records for 408. In these, we identified patient-reported wellness for 153 patients (38%). In 97 cases this was recorded in both medical and nursing notes, for 39 patients it was recorded in the nursing notes only and for 14 patients it was recorded in the medical notes only. In 87 patients this information was noted during the morning (5:00 am to 12:00) in 30 patients in the afternoon (12:00 to 17:00), the remaining were during evening and night hours.

In focus groups healthcare staff described multiple, fragmented documentation systems. Information about a patient's condition was spread across many different forms of documentation. Ward observation found that nurses would refer to the electronic nursing record, the paper based medical notes, the observation chart on the side of the patient's bed, a handover sheet, a safety brief, a ward map, a bay map, and “pocket notes”. During ward observations bedside charts were sometimes missing so that routine observations were written on scraps of paper, or paper towel, to add to the chart later.

Doctors in the focus group also reported not being able to find patient notes:

“It just takes me so long to see any patient this week because I can't find the notes” (Doctor)

Medical staff and nursing staff had parallel systems for documentation. Nurses spent much of their time transferring information from medical notes into the electronic nursing record. The electronic nursing record, inexplicably, could not be accessed by medical staff. The parallel systems made it difficult for doctors to access information about what had been observed overnight. While pain scores were routinely recorded, documentation of patient-reported wellness was scattered or absent. As a result, escalation decisions often depended on memory and informal communication rather than recorded trends.

#### 3.1.5 Escalation is shaped by hierarchy

As found in previous studies (27), escalation practices were strongly influenced by hierarchy. More junior doctors described reluctance to challenge senior decisions. Ward culture was identified as critical. Intensive Care, for example, was described as having a flatter hierarchy than some other medical specialties and a culture that encouraged escalation:

“Some departments might have more of a hierarchy where people might feel more scared to escalate things, but you know in [Intensive care ward] I feel like it is really good.” (Resident doctor)

#### 3.1.6 Patients and families as early warning systems

Staff recognised patients and families as valuable sources of early concern:

“The family is with her always so they will inform us if the patient needs anything. So if the patient is really agitated we can give her further medications.” (Nurse)

“You do find talking to a family member they might say “I think they might have an infection” like “they're not themselves”. “Not right” is another one. And you have a look and everything at that point looks ok and maybe two days later they start getting symptoms of a urine infection. The family called it before there was any evidence of it.” (Resident doctor)

The following is an extract from a focus group. It illustrates how doctors consider family concern as an indicator of deterioration, although family members” knowledge of the patient varied:

Resident 1: If I'm seeing someone in ED and I notice the family members are there with them I will try to speak to them while the family members are there
Resident 2: Or a family member walking up to you and saying they're worried.
Resident 3: Or if their families insist on waiting for like 8 h until I'm there to see them, that usually means there is something that they really, really want to tell me or get across to me.
Resident 4: I guess it's difficult with different family members escalating because we have patients with, like, family members abroad who are visiting, and their interpretation of the patient's deterioration is often different from a relative who has been living near

Family involvement was also shaped by environmental stressors and public narratives about NHS pressures which could create tension between the family and healthcare staff and hinder communication.

### 3.2 Assessing the feasibility of the patient wellness scale in everyday clinical settings

The sampling frame consisted of all patients on the four wards on four consecutive Friday afternoons (*N* = 448). Six patients declined to be part of the study (1%). Researchers asked each patient a verbal version of the Patient Wellness Scale. They obtained a response from 172 patients (38%). Reasons for not obtaining a response were dementia (*n* = 11), delirium (*n* = 16), absence from the bed side (*n* = 41), being asleep (*n* = 82), difficulty in hearing (*n* = 9), language barriers (*n* = 5), infection control (*n* = 44), clinically instability (*n* = 12), learning disability with no carer present (*n* = 2) or other reasons (*n* = 44). Of those who were willing and able to respond to the question, 98 (57%) were women. Most (118) were aged 70 years or over. The value of the National Early Warning Score (NEWS) at the time of interview and the highest value from the previous 24 h were charted. We found no association between responses to the Patient Wellness Scale in relation to improvement or deterioration and the direction of change in the NEWS (Chi-Square, *p* > 0.05).

Following the inclusion of the question as part of the electronic system for clinical monitoring, researchers observed staff interacting with the system as part of their everyday work across two wards on two consecutive Friday afternoons. During this time researchers observed observations taken on 40 patients. The Patient Wellness Scale was asked and documented for 22 patients, although one healthcare assistant completed the field on behalf of four patients but without asking the patient directly. In the remainder of patients, the observations were taken by temporary staff who were unaware of the change in practice and so simply ignored the new question. In one case the healthcare assistant recorded the observation on a piece of paper towel because the chart was missing.

## 4 Discussion

### 4.1 Key findings

Recording how patients are feeling is inconsistent and varies between wards and professional groups. Our findings suggest that staff already informally monitor subjective wellness all the time, but the hospital system is poorly configured to capture, communicate, integrate and act on that information reliably. The current documentation infrastructure limits reflexive monitoring and weakens the visibility of wellness as a longitudinal signal. A structured wellness measure could externalise and preserve knowledge that is currently lost with discontinuity. A formal wellness score could also function as a boundary object that legitimises information (28), supporting escalation across hierarchies. It could also provide a shared language for patient- and family-reported concern, reducing ambiguity and conflict. However, our study found weak evidence of feasibility of the Patient Wellness Scale and no demonstrated relationship with deterioration (at least in this dataset).

### 4.2 Interpretation in broader context

Our findings suggest that asking patients how they are feeling was perceived by staff as a routine and meaningful aspect of clinical practice. This suggests that implementation of the Patient Wellness Scale could generate coherence with healthcare staff that could support successful normalisation of the intervention in everyday work (29, 30). However, we also found limitations and our findings suggest there may be unintended consequences. When tested in a real-world clinical setting only a minority of patients found the question accessible. The subjective wellness of patients with dementia or delirium, hearing, speech or language challenges or very unwell may not be accurately captured by this form of monitoring. This raises the possible of potential health inequalities for vulnerable and critically ill patients.

Consistent with the broader implementation science literature (21), we found that the use and potential effects of the Patient Wellness Scale were shaped by organisational policies and routines. These reflect a broader cultural and political context that maintains a form of Taylorism in the division of nursing labour aimed at the efficient completion of tasks in a political context of resource constraints. In Wales important macroeconomic factors at this time were constrained health board budgets and subsequent downward pressure on recruitment to staff vacancies.

In our setting clinical monitoring was delegated to healthcare assistants. These were often temporary staff who were unfamiliar with other ward staff, equipment, routines and escalation protocols, indicating challenges for adoption and effectiveness. Delegating vital sign observations to Health Care Assistants is common practice in the NHS (31). A survey of nurses found that temporary staffing was a barrier to recognising and responding to clinical deterioration when there is a lack of knowledge and skills to calculate, record and interpret vital signs and escalate care (32). Previous research has also attributed the reduced adherence to the early warning system protocol on weekends to the use of temporary staff (33). Staff training is provided for permanent, rather than temporary staff (32). Lone working, or working with unfamiliar staff, can also reduce nurses' confidence is using their clinical skills (32). The current context of workforce shortages and use of temporary staff is therefore likely to limit the effectiveness of the intervention (34). It is well established in the safety literature that implementation of evidence-based interventions or changes to practice require clinical leadership, knowledge brokers, and ongoing education (30), however, the potential for these forms of facilitation to support implementation of the Patient Wellness Scale is limited by the large proportion of temporary staff involved in clinical monitoring.

Previous research has established that ward nurses aspire to make meaningful connections with patients, gain an in-depth knowledge of them as individuals, and include them and their families in decisions in a meaningful way (35–37). However, the organisational setting can severely restrict their capacity to build therapeutic relationships (35). Our study found that persistent staff shortages, combined with an expanding number of mandatory reporting requirements, resulted in a task-based approach to nursing, where the focus is on efficient completion of discrete tasks rather than responding to the individual needs of patients. Since the Francis report into serious failings in care at Mid Staffordshire hospital (38), there has been growing recognition of the way a task-based approach results in poor quality care (22). It is also a known safety risk that reduces nurses “awareness of patients” overall clinical condition (12).

To understand how a new tool brings about benefits it is important to understand the interaction of the tool with the user in particular contexts. Our study suggests that in the context of time pressure, temporary staffing, and a task-based approach to nursing the there is a risk that the Patient Wellness Scale will be implemented mechanistically. It will become “another thing to be done” rather than a meaningful way of including patients in the recognition and timely response to deterioration. This could increase the safety risk to patients if it replaces nurse-patient interaction and engagement.

In our setting nurses spent approximately 80% of their time on documentation. This limited their proximity to patients and opportunities to get to know the patient as an individual. It also limited their ability to establish a holistic understanding of the patient's condition and an awareness of how this developed over time. Much of this documentation related to organisational mandated risk assessments and quality improvement tools. Additive change means asking staff to do more and more and contributes to safety “clutter” (39). There is a risk that introducing the Patient Wellness Scale will add to the documentation burden on nurses while doing nothing to support the holistic understanding of the patient condition that supports safety. The Patient Wellness Scale, on its own, will not improve proximity and opportunity for nurses to get to know patients or reverse an impoverished nursing practice focused on the efficient completion of tasks.

The proportion of time that nurses spent on documentation was higher than that recorded previously in a time and motion study in an acute Trust in the NHS in Wales (40). The time and motion study found that nurses spent 10.6% of their time on “documentation”, although time spent on some forms of documentation was included in other categories, such as “admin and clerical”, “handover” and “observing physical symptoms” which makes comparison difficult. While there are a number of international time and motion studies of nursing work there are challenges when making comparisons due to differences in how documentation is measured. Most studies distinguish between “indirect” and “direct patient care” (41). Recent studies have found that, in acute hospitals, only 27 −32% of nurses' time is spent on direct patient care (41, 42). As international studies report from different funding systems this limits the applicability to NHS organisations. It would therefore be helpful for future research to calculate the total time nurses spend on all forms of documentation in the UK NHS.

Both doctors and nurses reported spending a significant amount of time looking for notes. When we came to do the analysis of patient records, we also found some files could not be located. We therefore recommend that future research captures the amount of time spent looking for notes and how this impacts of patient care.

Our study has limitations dictated by the framework of local service improvement: it is limited to a single site, focused on small-scale feasibility testing over a short time period, limited observation of the electronic implementation, and the absence of data on clinical outcomes.

Our study is across four different wards in one organisation. However, previous research suggests that the features of the organisational context (staff shortages, use of temporary staff and task-based nursing practice) and of clinical monitoring (incomplete, inaccurate and poor adherence to escalation protocols) are features of NHS acute hospitals more generally (33, 43).

### 4.3 Contribution to understanding of patient safety

This study contributes in four important ways:

1. It explains a known safety problem, rather than simply describing it: Many papers state that NEWS is insufficient or that “soft signs” matter (44). Our study shows how and why soft signs are currently used, lost, or overridden in real ward work, and under what conditions they influence escalation.
2. It reframes asking patients “how are you feeling?” as safety work: It is not just compassionate care, but active deterioration surveillance. That reframing positions patient-reported wellness as legitimate safety data, not an optional or “nice-to-have” add-on.
3. It makes the invisible workforce visible: By foregrounding health care assistants as central to deterioration recognition, our study adds empirical weight to long-standing but under-evidenced claims about their safety role (45). This has implications for training, staffing models, and escalation pathways.
4. It directly informs implementation of national policy: This study is timely and policy relevant. It speaks directly to Martha's Rule by identifying why escalation is hard, how hierarchy operates in practice, and what kinds of tools might realistically support earlier challenge.

### 4.4 Implications for clinical care

Detecting deterioration at an early stage remains a key priority in the NHS to avoid preventable deaths. Additionally, there is an increasing role for the patient and their family members to have in their own care but finding an effective way to involve them is still in works. Having a standardized way of recording patient-reported wellness could improve continuity of care, support patient-centred care, and enhance communication between staff and patients. Importantly, patients could feel empowered to let staff know about their concerns and wellness increasing the engagement in their care. While subjective wellness is an important indicator to confirm effectiveness of treatment and alert clinicians to failure of treatment and deterioration, the right format for the collection of this information and its translation into actionable data remains to be confirmed.

### 4.5 Implications for research

Larger studies are required to confirm how subjective wellness relates to metrics of physiological deterioration and in which patient groups, in which settings, this can be used to change diagnostics, treatment and outcomes. Furthermore, there is a need to determine if routine assessment and documentation of wellness lead to measurable improvements in patient physiology as measured by NEWS, mortality, morbidity, safety, satisfaction or other outcomes.

We have in the present work explored aspects of feasibility including workflow integration, but more detailed work will be required to explore reach, accessibility and implementation fidelity.

## 5 Conclusion

Information about patient wellness was inconsistently documented and fragmented across systems. Standardised approaches to recording patient- reported wellness may have the potential to support communication and continuity of care, but the right format for collection of this information and its translation into actionable data and clinical outcomes remains to be confirmed.

Incorporating patient and families' views on wellness could provide patients with a more holistic care that is individualized to their needs. However, the Patient Wellness Scale is not accessible to many vulnerable and critically ill patients. In the context of staff shortages, temporary staffing, and a task-based approach to care, there is a risk that the patient wellness scale will be implemented mechanistically, adding to the documentation burden on nurses while doing nothing to support the holistic understanding of the patient condition that supports safety.

## Data Availability

The raw data supporting the conclusions of this article will be made available by the authors, without undue reservation.
