# Supplementary material for: Recognising and responding to clinical deterioration in acute hospitals. Recording patient-reported wellness after Martha's rule
Source: Front Health Serv. 2026 Aug 26;6:1854717. doi: 10.3389/frhs.2026.1854717 (PMC13553874; doi:10.3389/frhs.2026.1854717)
Supplement: Supplementary file 1 [file Supplementaryfile1.docx]

| **Title** | **Recognising and responding to clinical deterioration in acute hospitals. Recording patient-reported wellness after Martha’s rule** |
| --- | --- |
|  |  |
| **Subbe**, Christian | Conceptualisation, Methodology, Writing original draft, Editing |
| **Jones**, Lorelei | Conceptualisation, Methodology, Analysis, Writing original draft, Editing |
| **Banfield**, Aimee | Methodology, Data collection, Analysis, Writing – review & editing |
| **Siji**, Sahla | Methodology, Data collection, Analysis, Writing – review & editing |
| **Munduchirayil Ajimon**, Lakshmipriya | Methodology, Data collection, Analysis, Writing – review & editing |
| **Phythian**, Catherine | Methodology, Data collection, Analysis, Writing – review & editing |
| **Kaba**, Adama | Methodology, Data collection, Analysis, Writing – review & editing |
